# The sternal wire code; Solving the problem of missing coronary artery bypass graft records during cardiac catheterization

**DOI:** 10.1016/j.ijcha.2018.04.004

**Published:** 2018-04-27

**Authors:** David S. Wald, Ben J. Wald, Angus Radford, Alex Shipolini

**Affiliations:** a Barts Heart Centre, St Bartholomew's Hospital, West Smithfield, London EC1A 7BE, United Kingdom; b Wolfson Institute of Preventive Medicine, Barts and The London School of Medicine and Dentistry, Queen Mary University of London, London EC1M 6BQ, United Kingdom; c Dulwich College, Dulwich Common, London SE21 7LD, United Kingdom

**Keywords:** CABG, Coronary angiography, Sternal wire code

## Abstract

**Background:**

The sternal wire code records details of coronary artery bypass surgery (CABG) inside patients, based on the orientation of wires used for sternal closure. Visible on X-ray, the code overcomes the problem of missing graft-notes needed before repeat angiography. We determined (i) the potential value (ii) acceptability and (iii) accuracy of the code in practice.

**Methods:**

(i) Consecutive coronary angiogram reports (2015–2016 Barts, London) were reviewed to identify patients with previous CABG and those with and without graft-notes before angiography. (ii) UK surgeons were surveyed on whether they would insert the code during CABG. (iii) A clinician, blinded to operative details, interpreted 16 post-CABG X-rays, 8 with the code and 8 without.

**Results:**

(i) Of 6483 angiography patients, 559 had previous CABG (9.2% (8.5–10%)). Graft-notes were missing in 91/559 (15.1% (12–18%)); almost all (88/91) among patients with acute myocardial infarction. (ii) In the survey, 66/71surgeons (93% (84–98%)) were willing to use the code. (iii) In the accuracy test, all coded X-rays were identified and 28/28 grafts correctly interpreted (*p* < 0.001).

**Conclusions:**

About 1 in 6 patients with previous CABG, who require emergency coronary angiography, undergo this procedure without graft-notes and would benefit from the sternal wire code which appears clinically acceptable and accurate.

## 1 Introduction

Coronary artery bypass surgery (CABG) is the most common operation carried out by cardiac surgeons [1,2]. Bypass grafts may occlude over time, resulting in recurrent angina or acute myocardial infarction. The management of such patients can be complicated and requires detailed knowledge of the previous surgery; how many grafts were implanted and whether they arise from the subclavian artery or aorta, to guide invasive angiographic imaging, assess graft patency and offer treatment by angioplasty or repeat CABG [3,4]. This information is not always available.

The sternal wire code is a method for permanently recording CABG details inside the patient [5]. It overcomes the problem of missing surgical records (graft-notes) when patients require repeat coronary angiography after CABG. Prior knowledge of the number and location of grafts reduces procedural complexity, radiation and contrast use [6,7] and is regarded as essential upfront information to minimise the risk of complications (stroke, heart failure and renal failure) and missing an occluded graft in need of intervention [[6], [7], [8]].

**Fig. 1:**
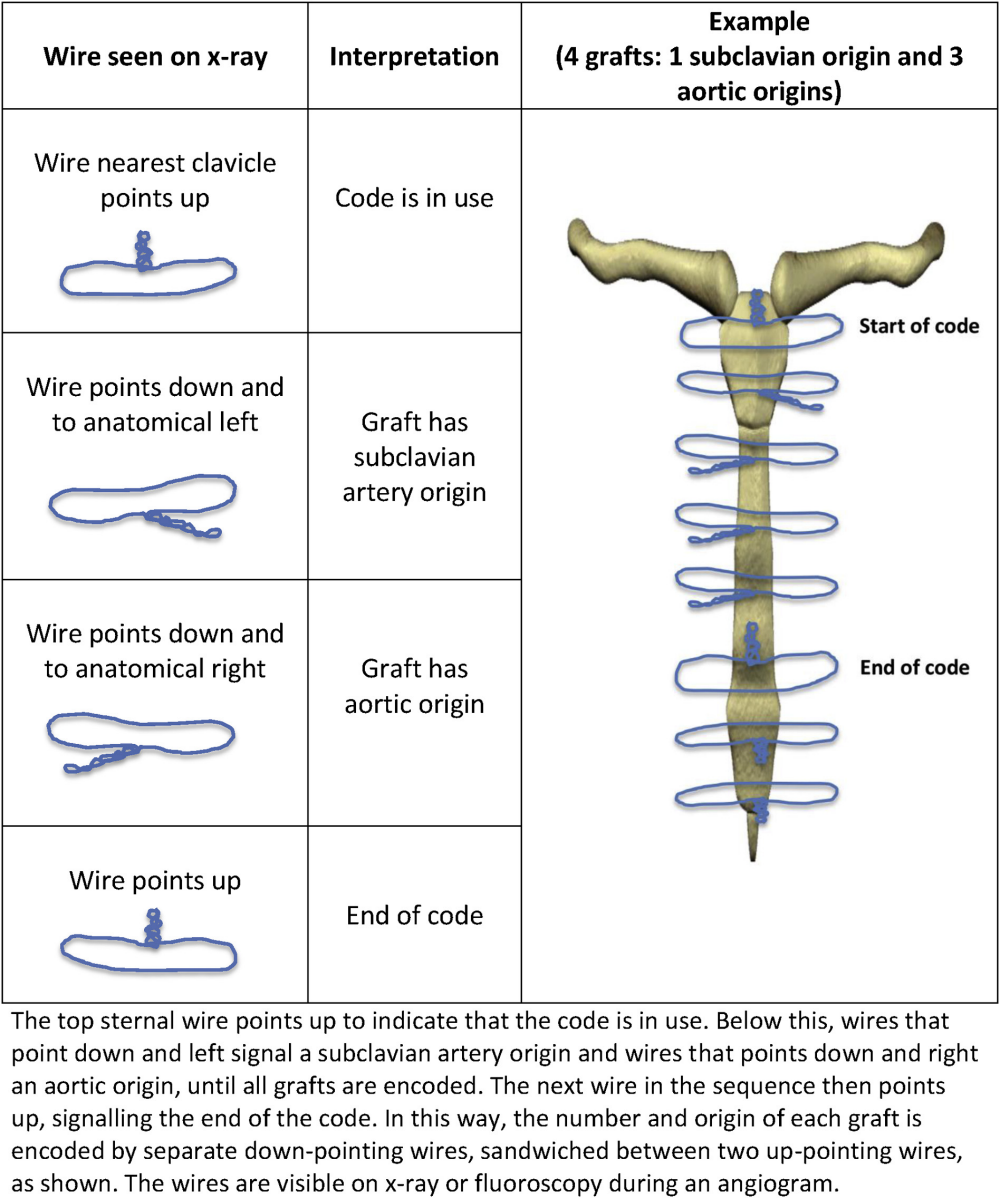
Sternal wire code – how it works.

The code is based on the orientation of the wires used to close the sternum after CABG (Fig. 1). It requires no new technology or surgical technique to implant and the wires are visible on fluoroscopy (X-ray) during an angiogram, providing the essential graft information when it is needed.

There is uncertainty over the proportion of patients who would benefit from such a code and the feasibility of introducing it into routine use. This prompted us to determine the prevalence of missing graft-notes in patients undergoing angiography and to assess the acceptability and accuracy of using the sternal wire code in practice.

## 2 Methods

### 2.1 Prevalence

We reviewed the electronic hospital records of 6454 consecutive patients undergoing coronary angiography at the Barts Heart Centre, London over a 1 year period (January 2015 to January 2016). From the procedure reports we identified patients who had undergone CABG and determined whether the graft-notes were available at the time of angiography. We determined whether the angiogram was required as an emergency procedure to treat a myocardial infarction or whether it was an elective investigation for stable angina. We also determined the number of patients who required additional imaging (aortography, computed tomography (CT) coronary angiography or repeat invasive angiography) because of uncertainty over whether all grafts had been seen during the initial angiogram.

### 2.2 Acceptability

The willingness of surgeons to use the sternal wire code during CABG was assessed by questionnaire among cardiac surgeons attending the United Kingdom's Society for Cardiothoracic Surgeon's 2017 annual meeting. A poster describing the code was presented and consecutive surgeons were asked whether they would, in principal be willing to orientate their sternal wires to encode the graft information for future doctors. Completed questionnaires were returned and the positive and negative responses counted.

### 2.3 Accuracy

The accuracy of interpreting the sternal wire code was assessed by examining the post-operative X-rays of 16 patients following CABG; 8 consecutive patients in whom the code had been used and 8 in whom it had not been used. A cardiologist, who was unaware of the operation details, was shown the 16 post-operative X-rays in random sequence and asked to identify the X-rays displaying the code and among them the number and origin of bypass grafts used. The observed record was compared with details from the operation notes.

Data were presented as absolute numbers and proportions together with 95% confidence intervals. *p* values were calculated using the binomial distribution. STATA was used for all analyses. The project was registered with the Clinical Effectiveness Board at Barts Health NHS Trust. Written consent was obtained from all patients whose X-ray images were used.

## 3 Results

Among 6484 patients undergoing coronary angiography, 599 had previous CABG (9.2% (95% CI 8.5–10%)) and 91/599 had no graft-notes available at the time of the angiogram (15.1% (12–18%)). In 87 of these 91 patients (96% (89–99%)), the angiogram was required as an emergency for an acute myocardial infarction. In 20/91 patients (22% (14–32%)) a second imaging investigation was required (8 aortograms, 10 CT coronary angiograms and 2 repeat invasive angiograms) before the graft information obtained was judged to be complete.

In the survey of cardiothoracic surgeons, 71 questionnaires were handed out and all were completed and returned. There were 66/71 (93% (84–98%)) surgeons who were, in principal, willing to use the sternal wire code in CABG surgery and 5 who were not willing to use it.

**Fig. 2:**
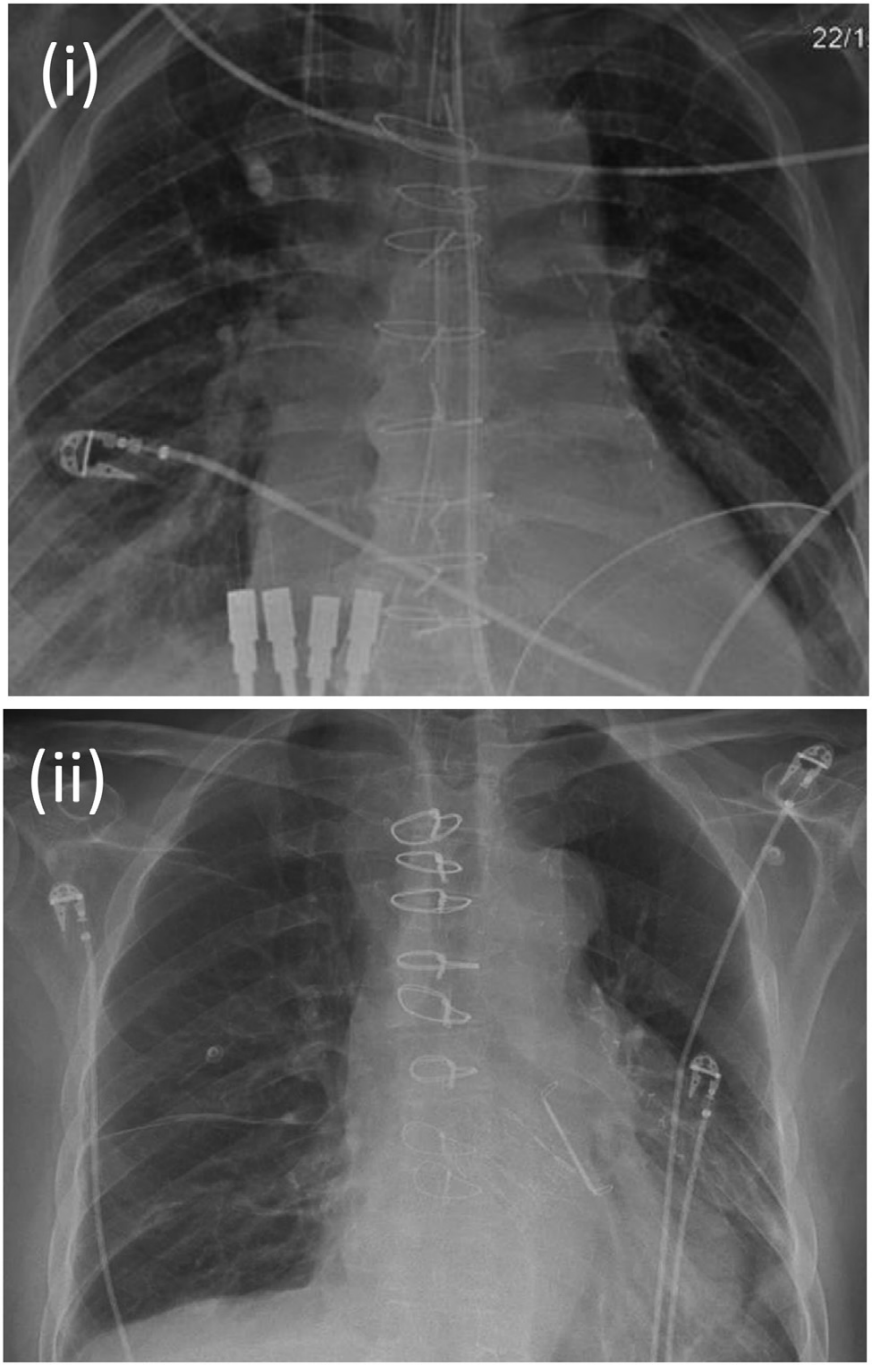
Example of (i) X-ray using sternal wire code (3 grafts, 1 from subclavian artery and 2 from aorta) and (ii) X-ray where code was not used.

Table 1 describes the characteristics of the 16 patients whose X-rays were examined to test the accuracy the code. There were no statistically significant differences between the 8 in whom the code was used and the 8 in whom it was not used. Overall the mean age of patient undergoing CABG was 70 years (range 54–83), 87% were male, 10 were elective procedures and 6 were urgent. Fig. 2 shows two examples of the 16 post-operative X-rays assessed in the accuracy test; one with the code and one without. The blinded assessor correctly identified the 8 X-rays with the code from the 8 without it (100%, *p* < 0.001). Out of a total of 28 bypass grafts encoded (3.5 grafts per patient), 28 were correctly interpreted (100%, *p* < 0.001).

**Table 1:** Characteristics of patients with and without sternal wire code whose postoperative X-rays were assessed in accuracy test.

	Code in use (*n* = 8)	Code not in use (*n* = 8)
Age^⁎^	73	68
Male	7	7
Smoker	4	3
Surgical details		
CABG alone	8	5
CABG + valve replacement	0	3
Emergency	2	4
Elective	6	4
Bypass graft details		
Subclavian origin, including	7	6
Left internal mammary	7	5
Right internal mammary	0	0
Aortic origin, including	8	8
One vein graft	0	2
Two vein grafts	4	3
Three vein grafts	4	3

Values are numbers of patients except ^⁎^median.

## 4 Discussion

Our results show that about 1 in 10 patients undergoing coronary angiography have had previous CABG and 1 in 6 of these patients did not have graft-notes available before their angiogram; the proportion who would benefit from use of the sternal wire code. Almost all of the patients without graft-notes (88/91) presented as emergencies with acute myocardial infarction, with little or no time to search for records that were not immediately available. Prior knowledge of the number and origin of surgical grafts is necessary before any angiogram [[6], [7], [8]] and is particularly important in patients with myocardial infarction to avoid missing an occluded culprit graft requiring angioplasty.

If our results are representative of those in other centres, then an estimated 4000 post-CABG angiograms are undertaken in the UK each year without grafts-notes (applying the 1 in 10 to the 240,000 angiograms performed each year and the 1 in 6 to those with previous CABG) [9]. The sternal wire code, if used routinely, has the potential to confer substantial benefit.

The prevalence of missing graft-notes and therefore, the potential value of the code in practice, may have been underestimated in our study, since it relied on operators specifically recording in the angiogram reports, that graft-notes were missing and this documentation may have been incomplete. It is also possible that increased use of electronic health records will, in the future, improve graft-note availability prior to angiography. Neither approach alone is likely to be sufficient; use of electronic records and the sternal wire code are complementary rather than alternative strategies.

### 4.1 Strengths and limitations

There are practical limitations to use of the code. Most surgeons use 8 wires to close the sternum, sufficient to encode up to 6 bypass grafts, but some operators (1 in 12 in our institution) use 4 longer double-loop wires sufficient for only two grafts. These variations in surgical practice will therefore limit the clinical application of the code. Also, between 0.2 and 5% of patients require wire removal because of discomfort or infection [10]. It is unlikely therefore that all patients who stand to gain from the code would, but even with only 50% uptake, about 2000 UK patients per year would benefit.

The code does not provide information on the distal course of a bypass graft (for example single, sequential or “y”) or its anastomosis (for example, to the right coronary artery or obtuse marginal coronary artery) but these variations do not limit the precision of the code in indicating the graft origin (subclavian or aorta) which is the most important information to the angiographer because this indicates how many grafts there are and where to find them.

False interpretation of the code is a concern. In the accuracy assessment, use of an up-pointing wire (which is non-standard) to signal the start and end of the code avoided the potential problems of falsely interpreting sternal wires when the code had not been used or miscounting the number of grafts implanted when it had been used. Our initial experience is therefore encouraging but the sample size is small, and a larger audited demonstration project would confirm whether the benefits of the code outweigh the risks of false interpretation. Most UK cardiothoracic surgeons (93% of the sample we surveyed) agreed, in principle, to use the code were it to be introduced. However, what is acceptable in principle may not translate into practice, and a demonstration project would also help to determine actual clinical uptake.

## 5 Conclusion

Missing graft-notes following CABG in patients undergoing coronary angiography is a significant problem, affecting about 1 in 6 patients, almost all of whom require emergency procedures. The sternal wire code is a simple solution that provides a permanent surgical record inside the patient. Early experience indicates that the code is accurate. A demonstration project is now needed to see whether surgeons will use it. The benefit to patients depends on wide clinical uptake, which is a significant implementation challenge.

## Contributors

BJW and DSW took part in the conception and design of the work. BJW, DSW, AR and AS took part in the acquisition of data, in the analysis/interpretation of data and in drafting the manuscript. All authors read and approved the final manuscript.

## Disclosure statement

DSW is a founder of Polypill Ltd. BJW, AR and AS declare that they have no competing interests.

## Funding

This research did not receive any specific grant from funding agencies in the public, commercial, or not-for-profit sectors.
